# Effect of naringin on gp120-induced injury mediated by P2X_7_ receptors in rat primary cultured microglia

**DOI:** 10.1371/journal.pone.0183688

**Published:** 2017-08-23

**Authors:** Qiang Chen, Hui Wu, Jia Tao, Chenglong Liu, Zeyu Deng, Yang Liu, Guoqiao Chen, Baoyun Liu, Changshui Xu

**Affiliations:** 1 Department of Physiology, Basic Medical College of Nanchang University, Nanchang, P.R. China; 2 The Second Clinical Medical College of Nanchang University, Nanchang, P.R. China; 3 The First Clinical Medical College of Nanchang University, Nanchang, P.R. China; Rutgers University, UNITED STATES

## Abstract

Human immunodeficiency virus type-1 (HIV-1) envelope glycoprotein 120 has been shown to activate microglia, causing release of inflammatory and toxic factors. The P2X_7_ receptor, primarily expressed on microglia, is closely associated with inflammation. Naringin, a plant bioflavonoid, has anti-inflammatory and anti-oxidative properties. We hypothesized that P2X_7_ receptor mediated gp120-induced injury in primary cultured microglia, and that naringin would have a protective effect. We showed that HIV-1 gp120 peptide (V3 loop, fragment 308–331) appeared to induce apoptosis of primary cultured microglia. However, there was a decrease of microglia apoptosis in gp120+naringin group compared with gp120 group. Using qPCR, Western blot, and immunofluorescence, we showed that gp120 stimulated expression of P2X_7_ mRNA and receptor protein, and this stimulation was inhibited by naringin. Treatment with gp120 increased concentrations of eATP, TNFα and IL-1β, and these effects were inhibited by naringin. Taken together, these results suggested that gp120 contributed to microglial cell injury and neurotoxic activity by up-regulating expression of P2X_7_, in a naringin-protective manner.

## Introduction

As the efficacy of antiretroviral therapy (ART) increases, the short-term mortality of acquired immune deficiency syndrome (AIDS) has decreased somewhat. AIDS is becoming a chronic disease with increased longevity [1]. The extended longevity of those living with HIV has led to a large number of diseases associated with inflammation, including cardiovascular disease, type II diabetes, cancer, and dementia [2]. The AIDS dementia complex (ADC), associated with both inflammatory and neurodegenerative processes, is a common complication of patients infected with HIV [3]. It is widely accepted that the infection and activation of microglia is closely related to neurologic dysfunction in HIV-associated dementia [4]. Envelope glycoprotein-120 is found on the surface of the HIV envelope, where it plays an important role in HIV’s binding to host cells and the subsequent inflammatory response [5]. It is important to note that brain macrophages and microglia, not neurons are susceptible to HIV-1 infection [6]. Studies have shown that brain macrophages and microglia infected by HIV-1 ultimately lead to neuronal injury and death [1,7]. Activated macrophages and microglia are proved to release neurotoxins including the pro-inflammatory cytokines tumor necrosis factor-α (TNFα) and interleukin-1β (IL-1β). These neurotoxins are thought to play critical roles in the mediation of neuronal injury and ADC pathogenesis [8].

P2X_7_ receptor, a purinergic receptor primarily expressed on macrophages and microglia, is associated with the release of inflammatory factors [9]. Activation of P2X_7_ receptor by extracellular ATP (eATP), released from injured cells, activates microglia and results in the release of inflammatory cytokines that are implicated in the pathogenesis of many neuronal disorders [10–12]. Activated microglia then amplify ATP stimulatory signals in an autocrine manner, leading to over-activation of microglia [13]. Taken together, these findings suggest that P2X_7_ receptor may represent a new target for the prevention of microglia injury and the treatment of ADC and other neuronal disorders associated with the inflammatory cascade.

Naringin is a type of plant bioflavonoid extracted mainly from grapefruit and other related citrus species. It possesses various biological and pharmacological properties including anti-inflammatory, anti-oxidative and anti-apoptotic properties [14,15]. In addition, naringin might mediate neuroprotective effects on Parkinson's disease [16], early brain injury and other neuronal disorders [17]. However, to our knowledge, there has been no report regarding a putative protective effect of naringin on gp120-induced primary cultured microglia injury.

Based on the fact that P2X_7_ receptor is closely related to inflammation, and that naringin exerts protective effect against inflammation and apoptosis, we hypothesize that the anti-inflammatory property of naringin may be related to inhibition of the P2X_7_ receptor. In this study, we explore the role of the P2X_7_ receptor in the pathogenesis of microglia injury, and we investigate the potential protective effect of naringin on gp120-induced primary cultured microglia injury.

## Materials and methods

### Primary microglia culture and grouping

This study was approved by the Basic Medical College of Nanchang University. Sprague-Dawley rats (1–2 days old) were obtained from the Laboratory Animal Science Department of Nanchang University. Animals were treated according to the guidelines for Care and Use of Animals set by the Ethical Committee of Nanchang University. Neonatal rats were anesthetized hypothermically using ether. Then they were fully disinfected with 75% alcohol under anesthesia, and decapitated while being placed on ice. Whole brain was dissected to obtain mixed glia cells. The meninges and blood vessels were removed carefully using ophthalmic forceps. Brain tissues were then minced using ophthalmic scissors, and enzymatically digested using 0.125% trypsin at 37°C for 60 min. Samples were separated by centrifugation at 1000 rpm for 10 min and filtered through a 70 μm cell strainer. The mixed cells were cultured on 25 cm^2^ flasks which had been coated with poly-l-lysine (PLL). Each flask contained 6 ml DMEM/F12 medium (Gibco, USA) which included 10% FBS (BI, Israel) and 1% PEN/Strep (Solarbio, China). Cell concentration was approximately 10^6^ cells/ml calculated by blood cell counting chamber. The cells were cultured in a constant-temperature incubator (Sanyo, Japan) containing 5% CO_2_ at 37°C. After having been cultured for 7–9 days, microglia were isolated from the mixed glia by gentle shaking [18]. Harvested microglia were inoculated into 6-well plates (coated with PLL) for RNA/protein extraction, or into 24-well plates containing coverslips (coated with PLL) at a density of approximately 10^5^ cells/ml. After 30 min, culture medium was changed to remove non-adhering cells.

In subsequent experiments, purified microglia were divided into four groups at random: control group (Ctrl); gp120 model group (gp120); naringin treatment group (gp120+naringin); and drug solvent control group (gp120+DMSO). After microglia were inoculated into plates, all groups apart from Ctrl group were incubated with 2.0 μg/L gp120 for 24 h. Gp120+naringin group was treated with 80 μM naringin (dissolved in DMSO), and gp120+DMSO group was treated with 0.08% DMSO. The concentration of naringin (Sigma Company, USA) that we used was based on our previous study [19]. Naringin and DMSO were applied simultaneously with HIV-gp120 peptide (V3 loop, fragment 308–331); gp120 of HIV-1 MN (CXCR4-preferring) was provided by Cellmano Biotech Limited, and we used gp120 to represent HIV-gp120 (V3 loop, fragment 308–331) in this paper.

### Primary cultured microglia purity identification

Immunofluorescence was performed to identify the purity of primary cultured microglia. Purified microglia were inoculated on coverslips that had been coated with PLL at a density of 10^5^ cells/ml in 24-well plates. After drug treatment for 24 h, microglia were washed three times in PBS and fixed with 4% paraformaldehyde (PFA) for 15 min at room temperature. After washing, microglia were permeabilized in 0.3% Triton X-100 for 10 min and blocked with 10% normal goat serum for 1 h. Cells were then incubated with CD11b primary antibody (CST, USA; 1:200 diluted in PBS) overnight at 4°C. After washing in PBS, microglia were incubated with FITC secondary antibody (Beijing Zhongshan Biotech CO.; 1:200 diluted in PBS) for 1 h. Cells were then incubated with DAPI (Boster, China) for 90 s for nuclear staining. Finally, preparations were mounted on slides and imaged with a fluorescence microscope (Olympus, Japan).

### TUNEL assay

TUNEL assay was performed to detect cell apoptosis using the In Situ Cell Apoptosis Detection Kit (Boster, China). Microglia were inoculated on coverslips that had been coated with PLL and treated with reagents. After 24 h, microglia were washed three times in PBS and fixed with 4% PFA for 30 min at 37°C. After washing three times with PBS, microglia were digested using Proteinase K (1:200 diluted in 0.01M TBS) at room temperature for 30 s and washed three times in 0.01M TBS. According to the manufacturer’s instructions, microglia were incubated with TUNEL reaction reagent, biotin-labeled anti-digoxin antibody, and SABC diluent. Microglia were then incubated with DAPI for nuclear staining, and finally mounted to the slides. Apoptotic cells were imaged by a fluorescence microscope.

### Detection of eATP

After isolation and purification, microglia were seeded on 24-well plates at a density of approximately 10^5^ cells/ml per well. Each group was set with three parallel wells. After reagent treatment had been applied for 24 h, the culture medium of each group was harvested to detect levels of eATP with ATPlite one step kit (PerkinElmer, USA). Lyophilized substrate solution was reconstituted using the appropriate volume of buffer. A vial of lyophilized ATP standard solution was reconstituted with water, and ATP standard solution was diluted into 1000, 500, 250, 125, 62.5, 31.2, 15.6, 7.8 pM for obtaining standard curve. For detection, we used 384-well opaque plates, with each well containing either 25 μl sample or ATP standard. Another 25 μl substrate solution was added to each well. After that, the 384-well microplate was shook for two minutes at 1,100 rpm. A multifunctional microplate reader (PerkinElmer, USA) was used to measure the luminescence of each group. The ATP content of each group was calculated using a standard curve according to manufacturer’s instructions. The DOI link of our protocol is http://dx.doi.org/10.17504/protocols.io.i9tch6n.

### Quantitative real-time PCR

Quantitative real-time PCR (qPCR) was performed to measure the expression of P2X_7_ mRNA. After extraction, total RNA was quantified by measuring OD value at 260 nm, followed by reverse transcription using 25 ng of total RNA. Each sample included an RNA specific control for the evaluation of potential contamination of genomic DNA. We used a SYBR^®^ Green detection system to quantify expression of P2X_7_ at mRNA level using the ABI PRISM^®^ 7500 Sequence Detection System (Applied Biosystems Inc., USA). PCR amplification was performed using 2 μl cDNA, with a total volume of 20 μl. Each sample was assayed in triplicate. The upstream and downstream primer sequences of the P2X_7_ were as follows: 5′-TTACGGCACCATCAAGTGGA-3′ and 5′-GCAAAGGGAGGGTGTAGTCG-3′, respectively, with the expected product size being 218 bp. The expression level of β-actin was chosen as a reference. Forward and reverse primer sequences (5′-TAAGGCCAACCGTGAAAAGATG-3′ and 5′-TGGTACGACCAGAGGCATAC-3′, respectively) of β-actin were used for amplification, and the expected product size was 110 bp. Cycling parameters were as follows: 94°C for 30 s to activate DNA polymerase; 40 cycles of 94°C for 5 s and 60°C for 30 s for amplification; 95°C for 15 s, 60°C for 1 min, and 95°C for 15 s to obtain the melt curve. The average threshold cycle (CT) value of P2X_7_ minus the average CT value of β-actin was the ΔCT value (ΔCT = CT target—CT reference). Results were calculated as follows: ΔΔCT = ΔCT test sample—ΔCT calibrator sample. Expression levels of P2X_7_ (RQ) were calculated using the following equation: RQ = 2^-ΔΔCT^.

### Western blot

After reagent treatment for 24 h, primary cultured microglia seeded on 6-well plates were lysed in RIPA buffer (Beyotime Biotechnology, China) for protein extraction. The composition of RIPA buffer was as follows: 50 mM Tris (pH 7.4), 150 mM NaCl, 1% NP-40, 0.5% sodium deoxycholate, 0.1% SDS, sodium orthovanadate, sodium fluoride, EDTA and leupeptin. The lysate was centrifuged at 12000 rpm for 5 min at 4°C. The supernatant was collected and preserved at -20°C for later use. The protein content of the samples was determined via the Lowry method. Samples containing equal amounts of protein (20 μg) were diluted with sample buffer, heated to 95°C for 10 min, and separated on SDS-polyacrylamide gels (10%) using an electrophoresis device (Bio-Rad, USA). Proteins were transferred onto PVDF membranes. A multifunctional gel imaging system was used to detect chemiluminescent signals. Band density was quantified using Image-Pro Plus 6.0 software (Media Cybernetics, USA). The primary antibodies were as follows: rabbit polyclonal anti-P2X_7_ (Alomone, Israel; 1:400 dilution), and β-actin (Beijing Zhongshan Biotech Co., 1:800 dilution). The secondary antibody was goat anti-rabbit/mouse IgG (Beijing Zhongshan Biotech Co.). Band densities were normalized with β-actin internal controls.

### Immunofluorescence

Immunofluorescence was performed to identify co-expression of P2X_7_ and CD11b in primary cultured microglia. Primary antibodies were rat anti-CD11b primary antibody (CST, USA; 1:100 diluted in PBS) and rabbit anti-P2X_7_ antibody (Alomone, Israel; 1:200 diluted in PBS). Secondary antibodies were goat anti-rabbit TRITC IgG and goat anti-mouse FITC IgG (Beijing Zhongshan Biotech CO.; 1:200 diluted in PBS). Other experimental steps were performed as described in the section of primary cultured microglia purity identification.

### Inflammatory index detection

After drug treatment for 24 h, the culture medium of each group was harvested to detect levels of TNFα and IL-1β via ELISA (Boster, China). A multifunctional microplate reader (PerkinElmer, USA) was used to measure the OD values of each group. TNFα and IL-1β contents were calculated using a standard curve according to manufacturer’s instructions. The experiment was performed as described previously [20].

### Statistical analyses

SPSS 21.0 (IBM, USA) was used to analyze the data. All results were expressed as mean ± SD. Statistical significance was determined by one-way analysis of variance (ANOVA), and *Fisher’s* Least Significant Difference (*LSD*) test was used for multiple comparisons. Significance was defined as *P* < 0.05.

## Results

### Primary cultured microglia purity identification

**Fig 1:**
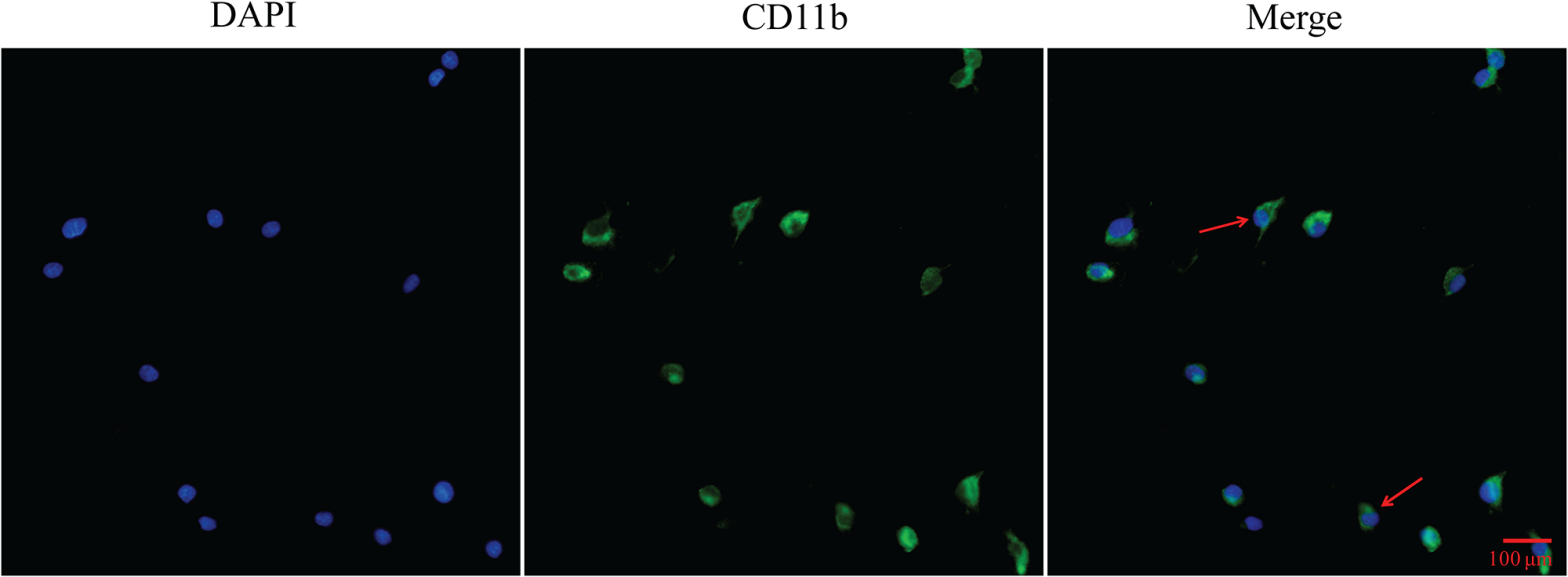
Primary cultured microglia purity identification by immunofluorescence. Blue signal indicates nuclear staining with DAPI; green signal represents CD11b staining with FITC; arrows represent microglia with typical morphology.

Prior to the performance of subsequent experiments, immunofluorescence was used to identify the purification degree of isolated primary microglia using the specific marker CD11b. By immunofluorescence, we showed that the purity of primary cultured microglia was greater than 90%. This level fully conformed to the requirements of subsequent experiments (Fig 1).

### Gp120 up-regulated expression of P2X_7_ mRNA and receptor protein in primary cultured microglia

**Fig 2:**
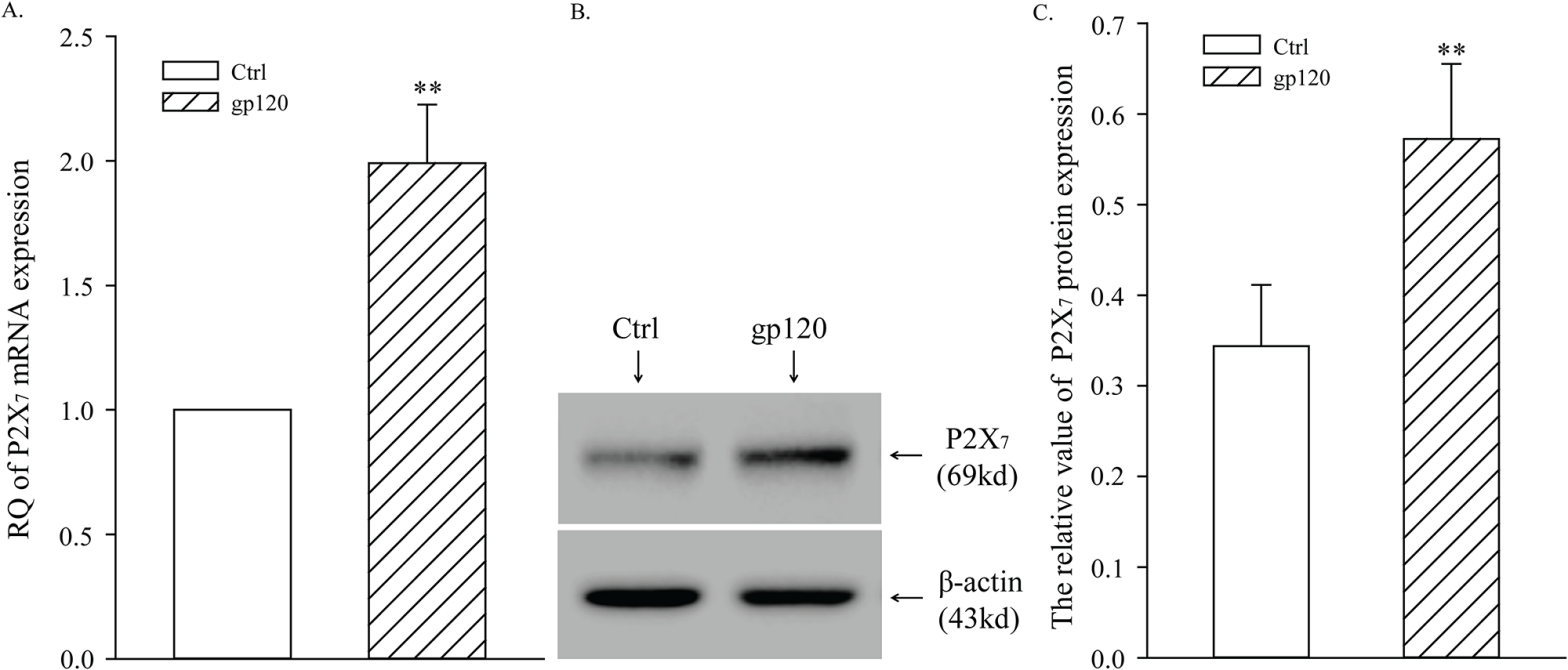
Gp120 up-regulated expression of P2X_7_ mRNA and receptor protein in primary cultured microglia. A, Relative expression of P2X_7_ mRNA detected by qPCR. B, SDS-PAGE electrophoresis of P2X_7_ receptor protein. C, Relative expression of P2X_7_ receptor protein; **P < 0.01, versus Ctrl group; *n* = 5.

In order to investigate a possible interaction between gp120 and P2X_7_ receptor expression in microglia, qPCR and Western blot were used to detect the mRNA and protein expression of P2X_7_. Compared with Ctrl group, expression of P2X_7_ mRNA and receptor protein in gp120 group was significantly up-regulated (*P* < 0.01), suggesting that gp120 up-regulated expression of P2X_7_ receptor in primary cultured microglia (Fig 2).

### Protective effect of naringin on primary cultured microglia apoptosis

**Fig 3:**
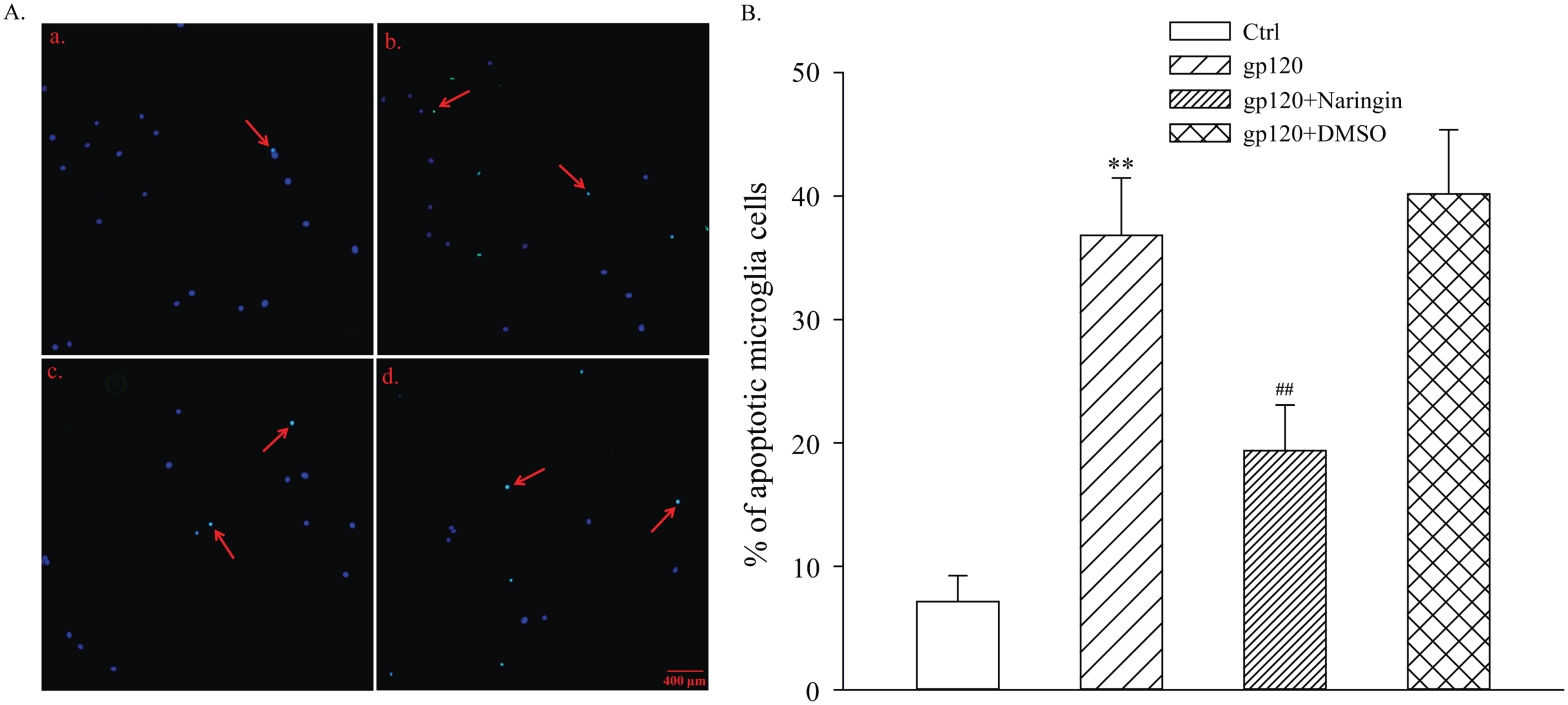
Primary cultured microglia apoptosis detected by TUNEL. A, Typical fluorescence image of apoptotic microglia in each group; blue signal indicates nuclear staining with DAPI; green signal represents TUNEL staining of fragmented DNA with FITC; arrows indicate apoptotic microglial cells; a, Ctrl group; b, gp120 group; c, gp120+naringin group; d, gp120+DMSO group. B, The percentage of apoptotic microglial cells in each group; **P < 0.01, versus Ctrl group; ^##^*P* < 0.01, versus gp120 group; *n* = 3.

After 24-hour reagent treatment, apoptosis of primary cultured microglia was determined by TUNEL assay. The percentage of apoptotic microglia (%) in Ctrl, gp120, gp120+naringin and gp120+DMSO groups was 7.15 ± 2.10, 36.81 ± 4.66, 19.37 ± 3.71 and 40.17 ± 5.20, respectively. Differences among groups were statistically significant (F_3,8_ = 42.858, *P* < 0.001). Compared with Ctrl group, apoptotic primary cultured microglia in gp120 group increased significantly (*P* < 0.01), suggesting that gp120 induced apoptosis of microglia. There was no apparent difference in microglia apoptosis between gp120 and gp120+DMSO groups (*P* > 0.05), suggesting that DMSO had no significant effect on primary cultured microglia apoptosis. There was a decrease in cell apoptosis in gp120+naringin group compared with gp120 group (*P* < 0.01), suggesting that naringin had an inhibitory effect on gp120-induced apoptosis in microglia (Fig 3).

### Naringin inhibited ATP release from primary cultured microglia

**Fig 4:**
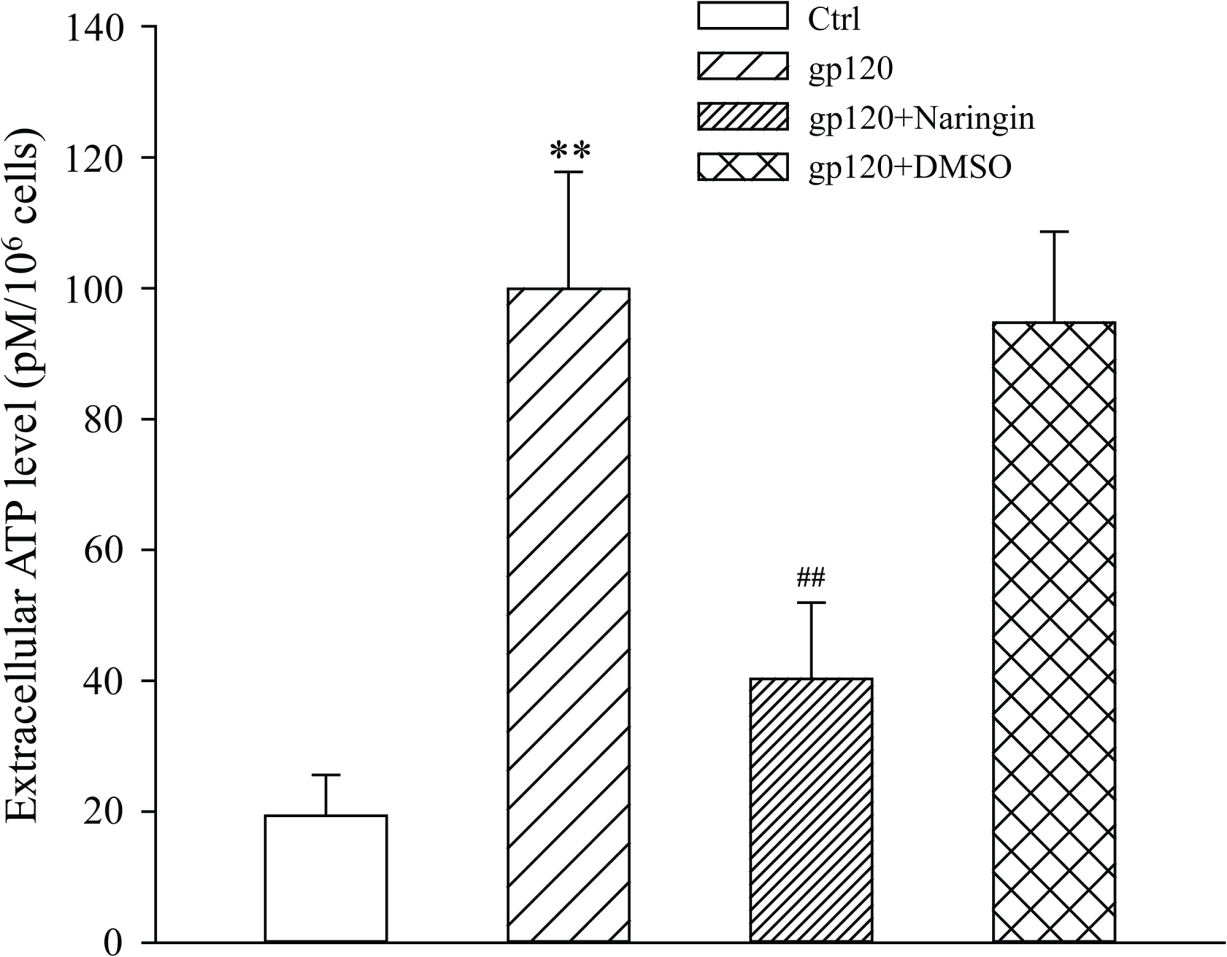
Concentrations of eATP in the supernatant of primary cultured microglia. **P < 0.01, versus Ctrl group; ^##^*P* < 0.01, versus gp120 group; *n* = 3.

To further explore the relationship among eATP, primary cultured microglia apoptosis and over-expression of the P2X_7_ receptor, we measured eATP content in the supernatant of each group using the ATPlite one step kit produced by PerkinElmer. Extracellular ATP concentrations (pM) of Ctrl, gp120, gp120+naringin and gp120+DMSO groups were 19.39 ± 6.25, 99.89 ± 17.86, 40.30 ± 11.66 and 94.73 ± 13.93, respectively. Differences among groups were significant (F_3,8_ = 27.810, *P* < 0.001). The contents of eATP in gp120 and gp120+DMSO groups were significantly higher than that of Ctrl group (*P* < 0.01). The concentration of eATP in gp120+naringin group was significantly lower than gp120 group (*P* < 0.01). There was no statistical significance between gp120+naringin and Ctrl groups with respect to ATP concentration (*P* > 0.05), suggesting that naringin may inhibit excessive release of ATP by injured cells, thus exerting a protective effect on gp120-induced primary cultured microglia injury (Fig 4).

### Naringin inhibited gp120-induced up-regulation of P2X_7_ mRNA

**Fig 5:**
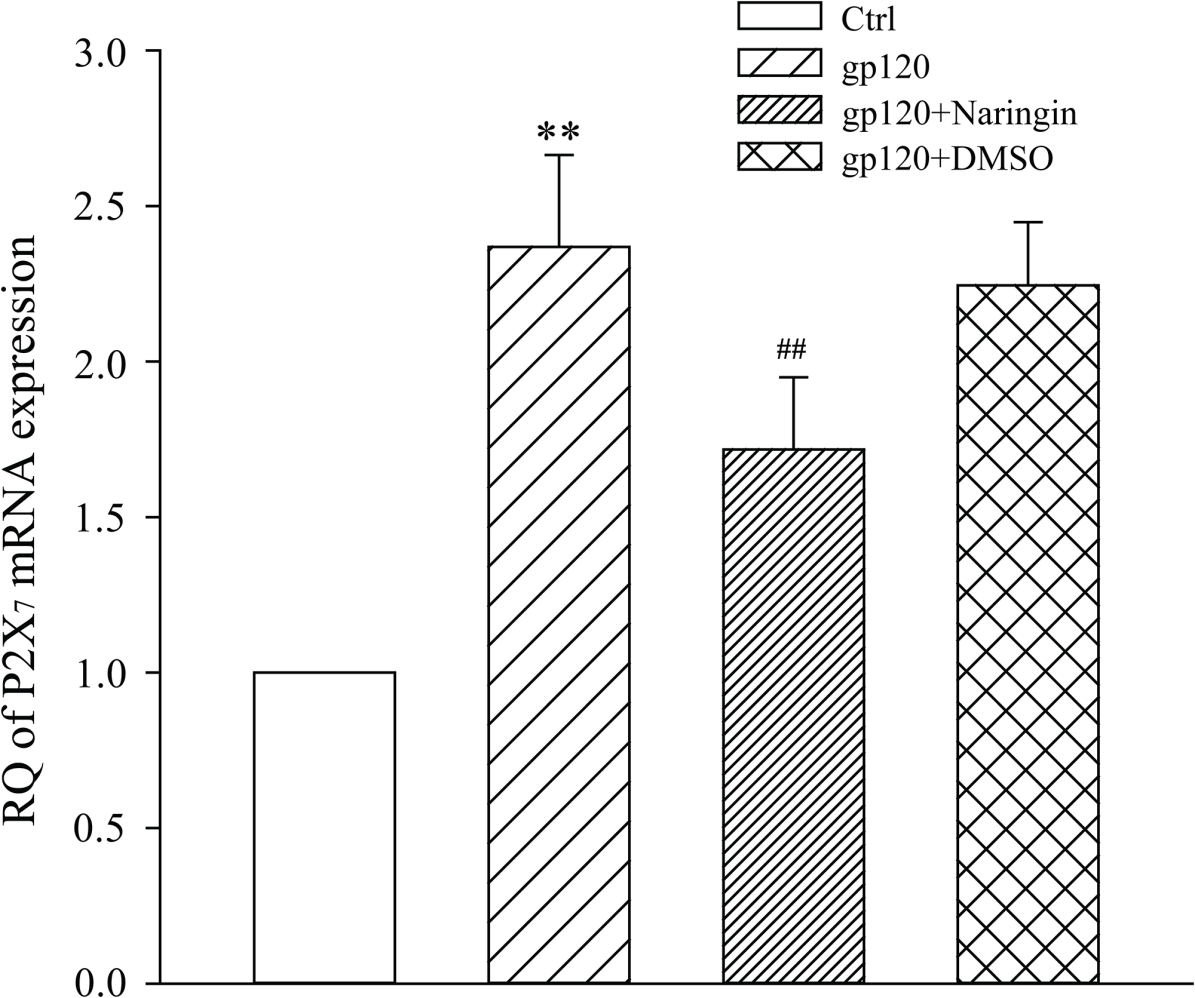
Expression of P2X_7_ mRNA in different treatment groups detected by qPCR. **P < 0.01, versus Ctrl group; ^##^*P* < 0.01, versus gp120 group; *n* = 5.

Quantitative PCR was used to measure the expression of P2X_7_ mRNA in primary cultured microglia. Relative expression of P2X_7_ mRNA in Ctrl, gp120, gp120+naringin and gp120+DMSO groups was 1.00 ± 0.00, 2.37 ± 0.30, 1.72 ± 0.23 and 2.25 ± 0.20, respectively. Differences among groups were statistically significant (F_3,16_ = 42.504, *P* < 0.001). Expression of P2X_7_ mRNA in gp120 group was significantly higher than that of Ctrl group (*P* < 0.01), suggesting that gp120 may promote up-regulation of P2X_7_ mRNA. There was no significant difference in P2X_7_ mRNA expression between gp120 and gp120+DMSO groups (*P* > 0.05). This suggested that DMSO had no significant effect on P2X_7_ mRNA expression in primary cultured microglia. Expression of P2X_7_ mRNA in gp120+naringin group was substantially lower than that of gp120 group (*P* < 0.05), suggesting that naringin had an inhibitory effect on P2X_7_ mRNA expression (Fig 5).

### Naringin reduced P2X_7_ receptor protein expression induced by gp120

**Fig 6:**
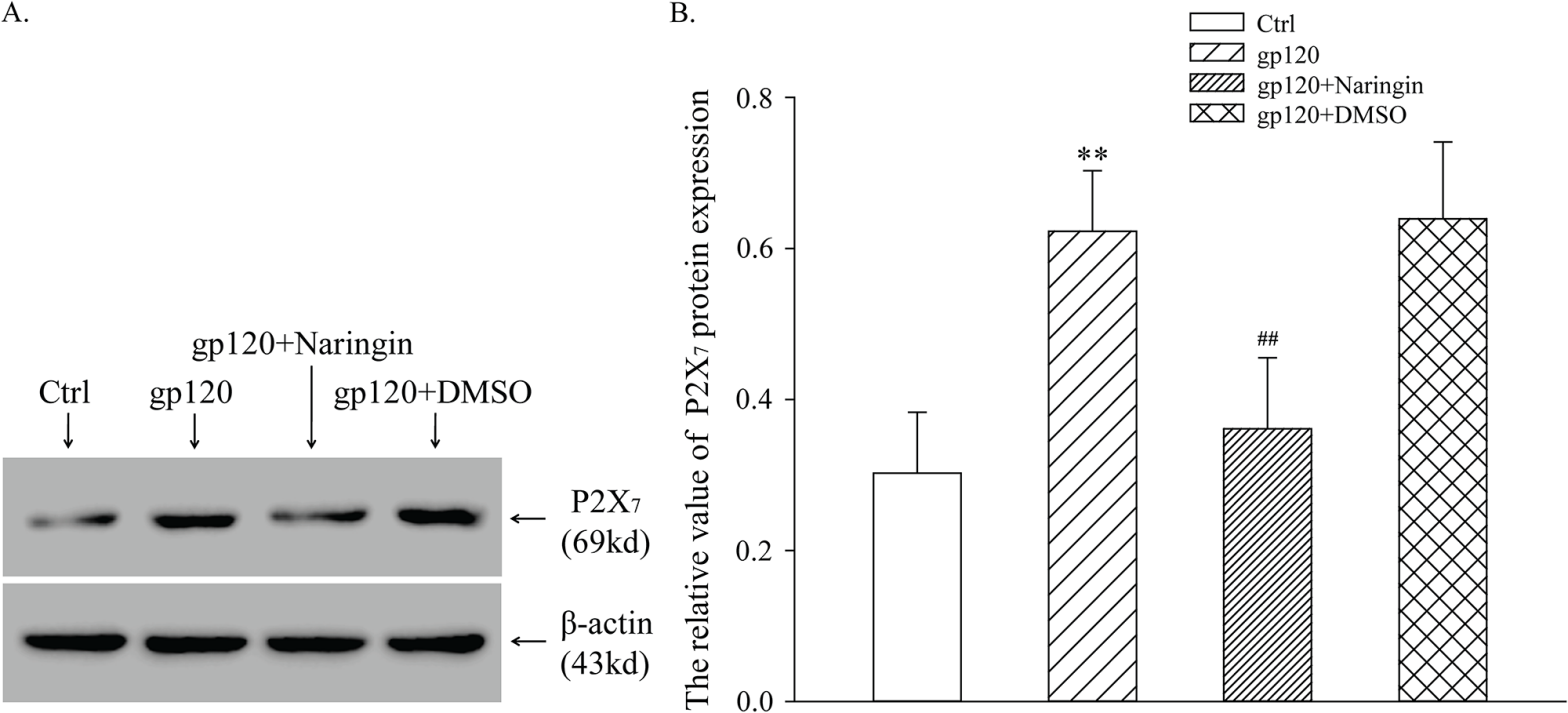
Expression of P2X_7_ protein measured by Western blot. A, SDS-PAGE electrophoresis of P2X_7_ receptor proteins in primary cultured microglia. B, Relative expression level of P2X_7_ receptor protein in primary cultured microglia; **P < 0.01, versus Ctrl group; ^##^*P* < 0.01, versus gp120 group; *n* = 5.

After extraction of total proteins, the expression level of P2X_7_ protein in primary cultured microglia was measured by Western blot. Relative expression of P2X_7_ receptor protein in Ctrl, gp120, gp120+naringin, and gp120+DMSO groups was 0.30 ± 0.08, 0.62 ± 0.08, 0.36 ± 0.09 and 0.64 ± 0.10 respectively. Differences among groups were statistically significant (F_3,16_ = 18.952, *P* < 0.001). Expression of P2X_7_ receptor protein in gp120 group was significantly higher than that of Ctrl group (*P* < 0.01), suggesting that gp120 up-regulated expression of P2X_7_ receptor protein. There was no significant difference in P2X_7_ protein expression between gp120 and gp120+DMSO groups, suggesting that DMSO had no significant effect on P2X_7_ protein expression. Expression of P2X_7_ receptor protein in gp120+naringin group was much lower than that of gp120 group (*P* < 0.01), suggesting that naringin inhibited gp120-induced up-regulation of P2X_7_ receptor protein expression in primary cultured microglia (Fig 6).

### Naringin reduced co-expression of CD11b and P2X_7_ receptor in primary cultured microglia

**Fig 7:**
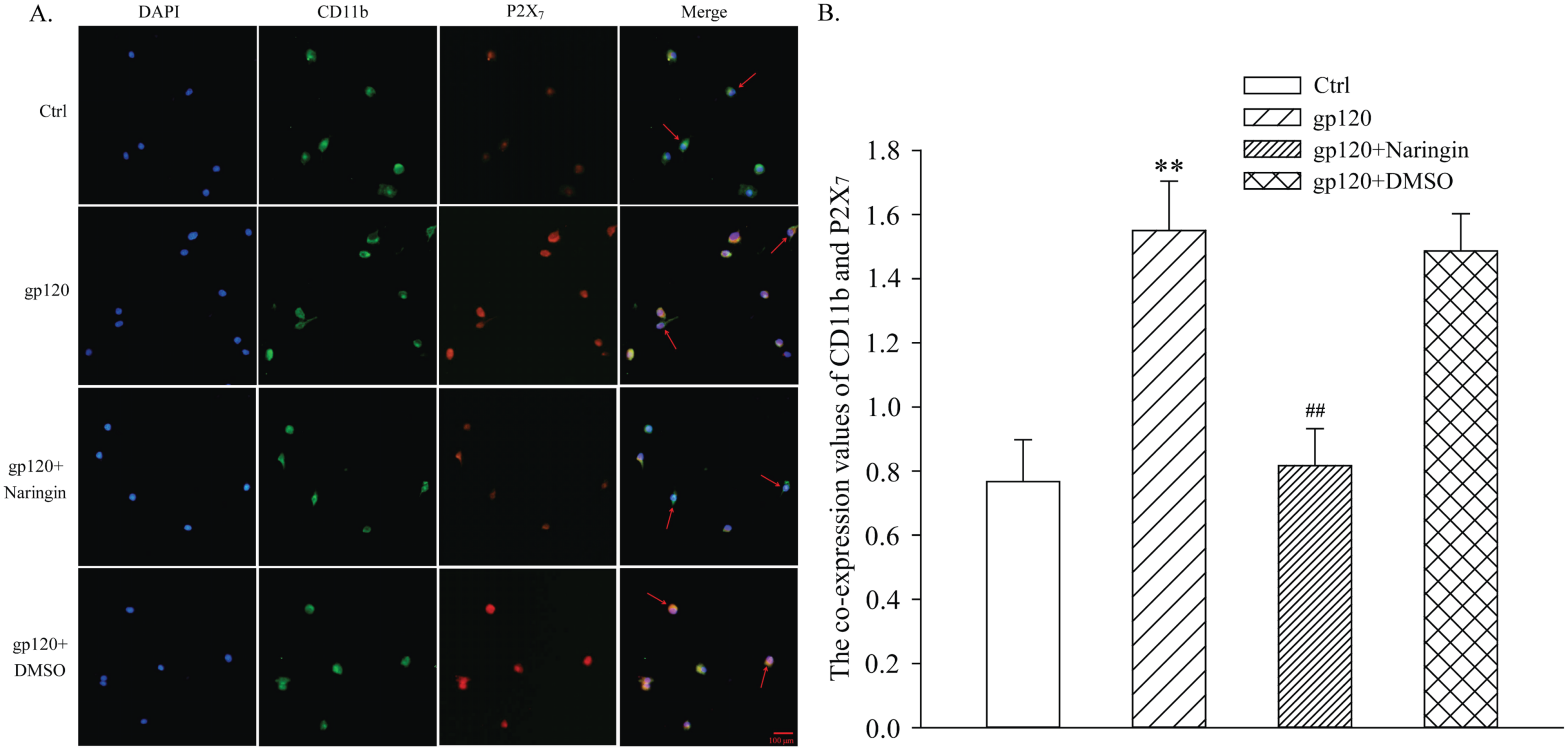
Co-expression of CD11b and P2X_7_ receptor in primary cultured microglia detected by immunofluorescence double labeling. A, Typical fluorescence image of primary cultured microglia; green signal represents CD11b staining with FITC; red signal indicates P2X_7_ staining with TRITC; Merge represents the P2X_7_ and CD11b double staining image; arrows represent microglia with co-expression of P2X_7_ receptor and CD11b. B, Co-expression values of CD11b and P2X_7_ receptor in each group; **P < 0.01, versus Ctrl group; ^##^*P* < 0.01, versus gp120 group; *n* = 3.

Immunofluorescence double-labeling was used to detect co-expression value of CD11b and P2X_7_ receptor in primary cultured microglia. Relative co-expression values of CD11b and P2X_7_ receptor in Ctrl, gp120, gp120+naringin and gp120+DMSO groups were 0.77 ± 0.13, 1.55 ± 0.15, 0.82 ± 0.12 and 1.49 ± 0.12, respectively. Differences among groups were statistically significant (F_3,8_ = 31.356, *P* < 0.001). Compared with Ctrl group, co-expression levels of CD11b and P2X_7_ receptor in gp120 and gp120+DMSO groups were higher (*P* < 0.01), while co-expression values of CD11b and P2X_7_ in naringin+gp120 group were markedly lower than those of gp120 group (*P* < 0.01). This suggested that naringin reduced expression of P2X_7_ receptor in primary cultured microglia induced by gp120 (Fig 7).

### Naringin inhibited release of TNFα and IL-1β induced by gp120

**Fig 8:**
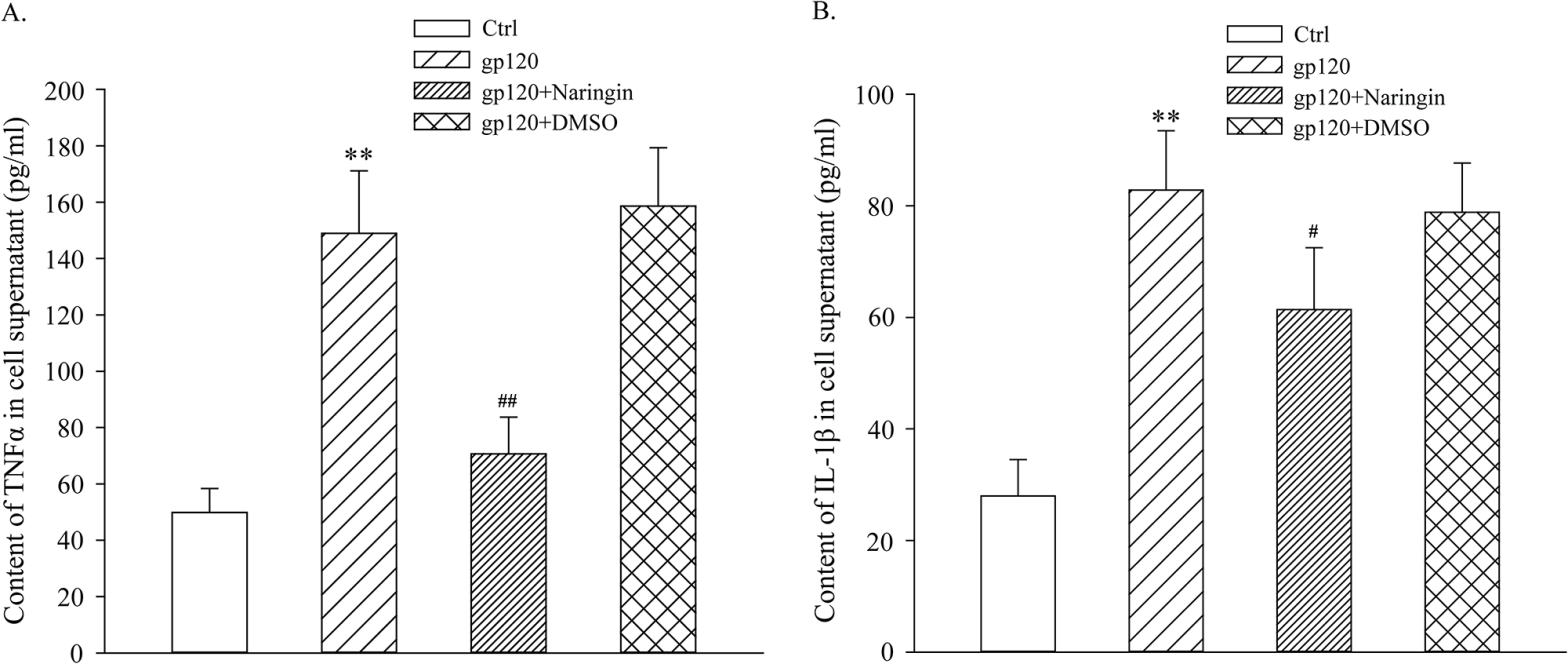
Concentrations of TNFα and IL-1β in the supernatant of primary cultured microglia. A, TNFα concentration in each group. B, IL-1β concentration in each group; **P < 0.01, versus Ctrl group; ^#^*P* < 0.05, ^##^*P* < 0.01, versus gp120 group; *n* = 3.

After OD values of each group were measured with a microplate reader, TNFα and IL-1β levels of primary cultured microglia were calculated. TNFα levels (pg/ml) in Ctrl, gp120, gp120+naringin and gp120+DMSO groups were 49.81 ± 8.50, 148.97 ± 22.14, 70.64 ± 13.07 and 158.61 ± 20.70, respectively. Differences among groups were statistically significant (F_3,8_ = 31.049, *P* < 0.001, Fig 8A). IL-1β concentrations (pg/ml) were: 27.98 ± 6.53, 82.83 ± 10.64, 61.40 ± 11.11 and 78.85 ± 8.82, respectively. Differences among groups were statistically significant (F_3,8_ = 20.982, *P* < 0.001, Fig 8B). TNFα and IL-1β levels in gp120 group were significantly higher than those of Ctrl group (*P* < 0.01), while there was no statistical difference between gp120 group and gp120+DMSO group (*P* > 0.05). This suggested that gp120 induced release of TNFα and IL-1β in primary cultured microglia. There was no statistical significance between gp120+naringin and Ctrl groups with respect to TNFα concentration (*P* > 0.05). Levels of TNFα and IL-1β in gp120+naringin group were lower than those of gp120 group, suggesting that naringin inhibited the release of TNFα and IL-1β induced by gp120.

## Discussion

As the major inflammatory cells in the central nervous system (CNS), microglia can be activated by HIV-1 envelope glycoprotein 120 through diverse mechanisms [21,22]. In a previous study, we showed that intra-cerebroventricular injection of gp120 caused learning and memory dysfunction of adult rats [23]. Using TUNEL assay, we found that the apoptosis rate of primary cultured microglia treated with gp120 increased considerably compared with that of Ctrl group. This suggested that gp120 induced primary cultured microglia apoptosis, and that this effect may probably be ascribed to prolonged oxidative stress, as described in a previous study [24]. Activated microglia amplify ATP stimulatory signals in an autocrine manner, leading to over-activation of microglia, followed by microglial cell apoptosis and uncontrolled inflammatory responses [25]. Naringin, a flavonone found in grapefruit, has been reported to have protective effect against neurodegeneration. Its anti-oxidative, anti-inflammatory and anti-apoptotic pathways may attenuate neuroinflammation [15,26]. In our study, the apoptosis rate of primary cultured microglia in naringin treatment group was lower than that of gp120 group, suggesting that naringin inhibited apoptosis of microglia induced by gp120. The apoptosis rate of microglia in the presence of naringin was significantly different from that of Ctrl group. We did not observe a completely restorative effect of naringin on gp120 induced microglia injury, suggesting that naringin may only partially prevent gp120-induced microglia injury mediated by the P2X_7_ receptor. A previous study demonstrates that naringin induces partial neuroprotective effects in a mouse model of Parkinson's disease *in vivo*, but fails to reverse the disease completely [16].

P2 purinergic receptors, whose ligands are extracellular adenine nucleotides (such as ATP) are important for modulating the inflammatory functions of microglia [27]. As a member of P2 purinergic receptor family, P2X_7_ receptor has been shown to play a crucial role in regulating the activity of microglia [12,28]. Under pathophysiological conditions, apoptotic or injured cells may actively or passively release nucleotides into the extracellular space. One study suggests that ATP released by gp120-injured or apoptotic cells may activate microglia by binding with P2X_7_ receptors [29], resulting in the processing and secretion of pro-inflammatory cytokines [30]. Another study demonstrates that prevention of over-activation of microglia by ATP may confer neuroprotective capacity in the context of both acute and chronic CNS diseases [31]. It is demonstrated that microglial cells expressing the P2X_7_ receptor are exquisitely sensitive to ATP-mediated cytotoxicity. In addition, eATP induces microglia apoptosis via the activation of P2X_7_ receptor [32,33]. Here, we demonstrated that the concentration of eATP in gp120 group was significantly higher than that of Ctrl group, suggesting that gp120 induced ATP release from primary cultured microglia. This release may activate the P2X_7_ receptor, resulting in microglial apoptosis. A possible mechanism may be that activation of P2X_7_ by ATP gives rise to influx of small cations, including Ca^2+^, Na^+^, and K^+^. This influx may lead to a series of cell-specific downstream signaling events, including formation of reactive oxygen species (ROS) and reactive nitrogen species, and either cell proliferation or cell death [34]. The concentration of eATP in gp120+naringin group was significantly lower than that of gp120 group. This effect may be attributed to naringin inhibiting primary cultured microglia apoptosis and reducing ATP release.

The P2X_7_ receptor, expressed at high levels on immune cells, has a more defined role than other purinergic receptors. For example, the P2X_7_ receptor triggers release of cytokines [35]. Activated microglia may express P2X_7_ receptors abundantly [36], leading to a cascade reaction that further augments the release of inflammatory factors. In our study, expression of P2X_7_ mRNA and receptor protein in primary cultured microglia was significantly higher in gp120 group than Ctrl group, while expression of P2X_7_ mRNA and receptor protein in naringin+gp120 group was markedly lower than that of gp120 group. Considering the fact that the apoptosis rate increased considerably and the P2X_7_ receptor was expressed abundantly in gp120 group, we may infer that gp120 induced apoptosis of primary cultured microglia, leading to abundant ATP release, which in turn robustly activated microglia and increased expression of P2X_7_ receptor. We confirmed our results with immunofluorescence. We showed that co-expression of CD11b and P2X_7_ receptor in gp120 group was significantly higher than that of Ctrl group, and Naringin inhibited this up-regulation. Taken together, these results suggested that gp120 up-regulated P2X_7_ mRNA and receptor protein, on which naringin had an inhibitory effect. This may be attributed to the fact that naringin inhibited excessive activation of microglia.

It is demonstrated that over-activated microglia overproduce a variety of pro-inflammatory cytokines, including IL-1β, TNFα and chemokines, all thought to give rise to ADC [37]. One study suggests that exposure of microglia to gp120 results in the activation of the inflammasome and associated IL-1β secretion [38]. Inflammatory cytokines produced by activated macrophages or microglia lead to neuronal injury and neurotransmitter function interference, resulting in deficits of learning and memory abilities [39]. Here, we showed that TNFα and IL-1β levels in gp120 group were significantly higher than those of Ctrl and gp120+naringin groups, suggesting that gp120 up-regulated the release of inflammatory factors, and that naringin inhibited this release. Though neurons themselves are not susceptible to HIV infection, neurotoxins such as TNFα and IL-1β secreted by infected and/or activated macrophages, microglia, and astrocytes may mediate neuronal injury [40]. Hyperactivation of microglia, together with subsequent release of inflammatory factors contributes to neuronal inflammation and death.

## Conclusion

In summary, we provide evidence that naringin exerts a partially protective effect on gp120-induced injury in primary cultured microglial cells, and the effect is most likely mediated by the P2X_7_ receptor. We hope that our results may facilitate the further study of HIV-1-related neurodegenerative diseases, and help improve the quality of life of those suffering from these diseases.

## Supporting information

S1 File Minimal data set. (XLS)
